# Draft Genome Sequences of *Streptococcus pyogenes* Strains Associated with Throat and Skin Infections in Lebanon

**DOI:** 10.1128/genomeA.00358-14

**Published:** 2014-05-15

**Authors:** Sima Tokajian, Jonathan A. Eisen, Guillaume Jospin, David A. Coil

**Affiliations:** a Lebanese American University, Department of Natural Sciences, School of Arts and Sciences, Byblos, Lebanon; b University of California Davis Genome Center, Davis, California, USA

## Abstract

We present the draft genome sequences of nine clinical *Streptococcus pyogenes* isolates recovered from patients suffering from sore throat and skin infections. An average of 2,454,334 paired-end reads per sample were generated, which assembled into 21 to 198 contigs, with a G+C content of 38.4 to 38.5%.

## GENOME ANNOUNCEMENT

*Streptococcus pyogenes* causes disease states that vary from mild sore throat to the flesh-eating disease necrotizing fasciitis (1). Necrotizing fasciitis is caused by group A toxin-producing *S. pyogenes* strains, known as “flesh-eating bacteria” (2). The *S. pyogenes* M protein (encoded by the *emm* gene) is used as an epidemiological marker based on the variability in the N-terminal end (3). The occurrence of *emm* types among group A streptococcus (GAS) isolates varies according to the geographical location (4). In this study, we selected different common *S. pyogenes emm* types for genome sequencing (SP1, *emm*12; SP2, *emm*108; SP3, *emm*89; SP4, *emm*28; SP5, *emm*1; SP6, *emm*89; SP7, *emm*22; SP8, *emm*85; and SP10, *emm*118). The chosen isolates were recovered from patients at the American University of Beirut Medical Center (AUB-MC) suffering from sore throat (types SP1, SP3, SP4, SP6, SP7, SP8, and SP10) and skin infections (types SP2 and SP5).

DNA was extracted (50 ng/sample) using the NucleoSpin tissue kit (Macherey-Nagel, Germany) and prepared for sequencing with the use of the Nextera XT DNA sample prep kit (Illumina). The samples were pooled together and then sequenced on an Illumina MiSeq for paired-end 250-bp reads. An average of 2,407,766 paired-end reads per sample were generated. Quality trimming and error correction of the reads resulted in an average of 1,951,506 high-quality reads. Sequence processing and assembly were performed using the A5 assembly pipeline. This pipeline automates the processes of data cleaning, error correction, contig assembly, scaffolding, and quality control (5). The initial assembly produced the following: for SP1, 171 contigs contained in 156 scaffolds; SP2, 34 contigs contained in 29 scaffolds; SP3, 32 contigs contained in 25 scaffolds; SP4, 48 contigs contained in 39 scaffolds; SP5, 40 contigs contained in 26 scaffolds; SP6, 23 contigs contained in 19 scaffolds; SP7, 160 contigs contained in 158 scaffolds; SP8, 186 contigs contained in 186 scaffolds; and SP10, 17 contigs contained in 12 scaffolds. During scaffolding, some contigs were merged based on short overlaps and read-pair information, yielding a reduced final collection of contigs that were submitted to GenBank for each sample. The final draft genome sequences consist of 187 contigs for SP1, including a combined 1,931,785 bases; 28 contigs for SP2, including a combined 1,724,470 bases; 29 contigs for SP3, including a combined 1,745,655 bases; 40 contigs for SP4, including a combined 1,906,357 bases; 34 contigs for SP5, including a combined 1,812,035 bases; 24 contigs for SP6, including a combined 1,733,948 bases; 170 contigs for SP7, including a combined 1,959,254 bases; 198 contigs for SP8, including a combined 1,924,015 bases; and 21 contigs for SP10, including a combined 1,773,604 bases. The G+C contents of the chosen isolates ranged between 38.4 and 38.5%.

### Nucleotide sequence accession numbers.

These whole-genome shotgun projects have been deposited at DDBJ/EMBL/GenBank under the accession no. AYPA00000000 (SP1-LAU), AWOZ00000000 (SP2-LAU), AWPA00000000 (SP3-LAU), AWPB00000000 (SP4-LAU), AWPC00000000 (SP5-LAU), AWPD00000000 (SP6-LAU), AWPE00000000 (SP7-LAU), AWPF00000000 (SP8-LAU), and AWPG00000000 (SP10-LAU). The versions described in this paper are the first versions, accession no. AYPA01000000, AWOZ01000000, AWPA01000000, AWPB01000000, AWPC01000000, AWPD01000000, AWPE01000000, AWPF01000000, and AWPG01000000, respectively.
